# Origin and consequences of silicate glass passivation by surface layers

**DOI:** 10.1038/ncomms7360

**Published:** 2015-02-19

**Authors:** Stéphane Gin, Patrick Jollivet, Maxime Fournier, Frédéric Angeli, Pierre Frugier, Thibault Charpentier

**Affiliations:** 1 CEA, DEN, DTCD, SECM, F-30207 Bagnols-sur-Ceze, France; 2 CEA, DSM, IRAMIS, NIMBE, UMR 3299, F-91191 Gif-sur-Yvette, France

## Abstract

Silicate glasses are durable materials, but are they sufficiently durable to confine highly radioactive wastes for hundreds of thousands years? Addressing this question requires a thorough understanding of the mechanisms underpinning aqueous corrosion of these materials. Here we show that in silica-saturated solution, a model glass of nuclear interest corrodes but at a rate that dramatically drops as a passivating layer forms. Water ingress into the glass, leading to the congruent release of mobile elements (B, Na and Ca), is followed by *in situ* repolymerization of the silicate network. This material is at equilibrium with pore and bulk solutions, and acts as a molecular sieve with a cutoff below 1 nm. The low corrosion rate resulting from the formation of this stable passivating layer enables the objective of durability to be met, while progress in the fundamental understanding of corrosion unlocks the potential for optimizing the design of nuclear glass-geological disposal.

## Abstract (web summary)

The long term durability of silicate glasses is of significant importance, not least due to applications in nuclear waste repositories. Here, the authors study glass corrosion and show that its rate drops as a passivating layer forms via a self-healing mechanism.

## Graphical abstract

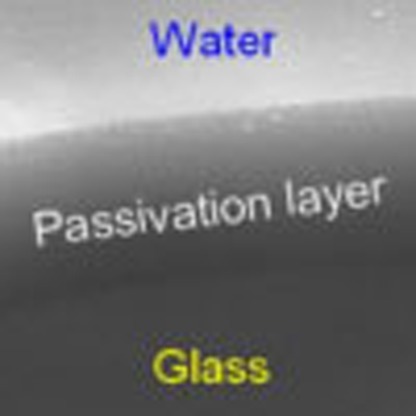

The behaviour of silicate glasses over geological timescales raises challenging scientific issues. One of the most prominent topics with a high social impact is the safe management of highly radioactive wastes originating from spent nuclear fuel reprocessing that, among others, depends on our capacity to predict the long-term corrosion rate of borosilicate glass^1^,^2^,^3^. Geochemists face similar fundamental problems with basaltic glass when calculating the chemical mass balance of the oceans^4^ or when attempting to assess potential CO_2_ sequestration by silicate rocks^5^. Current rate laws still cannot accurately predict the long-term behaviour of synthetic or natural silicate glasses over geological timescales mostly because of the existence of surface layers between pristine glass and bulk solution that both affect the transport of reactive species and change fluid properties at the glass surface compared with that of the bulk^6^,^7^. Surface layers are made of amorphous and crystalline metastable phases, formed by *in situ* condensation and precipitation reactions from aqueous species^8^. Glass dissolves following the Ostwald rule of stages, forming a series of alteration products progressively evolving towards thermodynamically stable phases^6^,^9^. Depending on the glass composition and on the conditions (for example, temperature, pH, solution composition and flow rate), this transformation into stable compounds can vary from hours to millions of years^6^,^10^,^11^!

In this study, glass corrosion mechanisms were investigated from macroscopic to atomistic scale, through isotopically tagged corrosion experiments performed with international simple glass (ISG)—a six-oxide borosilicate glass used as a reference material by the nuclear glass community^1^—and the use of complementary analytical techniques to provide evidence of the long-term rate-limiting mechanisms. The main experiment was performed with 16 coupons of ISG glass (with isotopes at the natural abundance) altered for 1 year in a static mode at 90 °C, pH_90 °C_ 7, in a solution initially saturated with amorphous ^29^SiO_2_ (Supplementary Fig. 1). Previous experiments showed that when altered in deionized water the leaching solution of ISG glass eventually reaches the equilibrium with amorphous silica (SiO_2am_) but this requires several years^12^. This starting solution thus enables the first transient stages of glass corrosion to be bypassed and focus placed on the processes governing the long-term rate. In parallel, a similar experiment was conducted with glass powder until its complete alteration, in order to examine the structural changes within the silicate network. The choice of pH 7 was motivated by two reasons. First, after similar investigations carried out at pH 9 and 11.5, it was found that results at pH 7 were the most convincing because glass corrodes about four times faster at pH 7 than at pH 9, although the fundamental processes are the same^13^. This led to larger amounts of material available for in-depth characterization. Second, a pH of 7 is typical of many natural waters; for instance, the water in equilibrium with the claystone studied in France for the storage of nuclear wastes is pH 7.3 at room temperature^14^. Together, these studies improve our understanding of silicate glass corrosion processes, by linking dissolution kinetics and structural modifications following water ingress into the solid. At this pH, the glass is passivated by a self-healing and poorly hydrated silica layer in which water is trapped in subnanometric pores. Conclusions can be extended at least until pH 9.5, leading to general recommendations for the safe management of highly radioactive waste glass.

## Results

### Glass still corrodes despite silica saturation conditions

**Figure 1:**
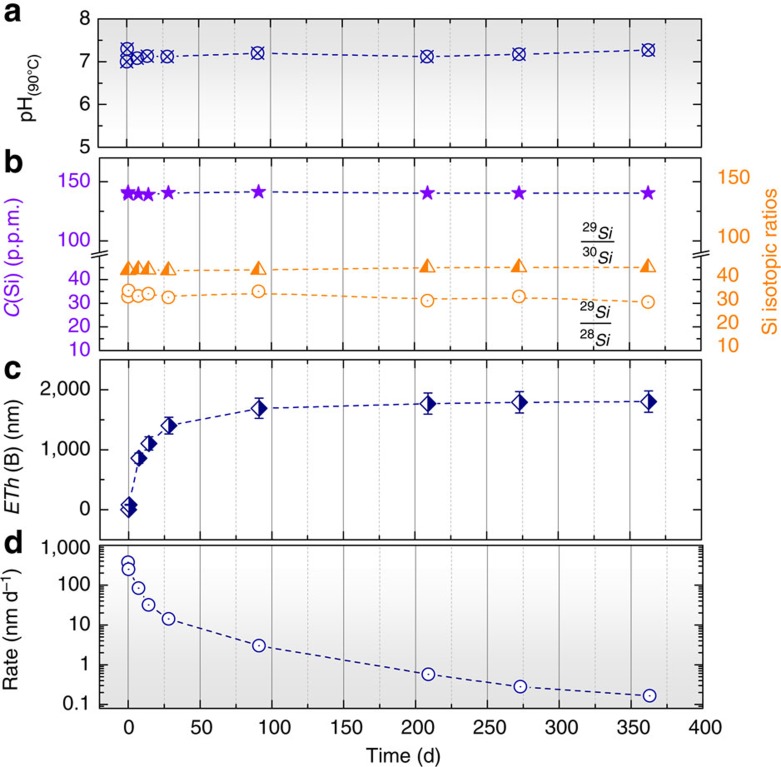
Data from solution analyses. Time evolution of the solution pH (**a**), the concentration of Si, ^29^Si/^28^Si and ^29^Si/^30^Si ratios (**b**), the equivalent thickness of altered glass (*ETh*) calculated from B release into solution (**c**) and the glass dissolution rate (**d**). The pH_90 °C_ was maintained at 7±0.25 by regular additions of diluted HNO_3_. Boron was used to calculate glass corrosion thickness and rate. This element is known to be a good corrosion tracer as it is very soluble and only slightly retained in the alteration layer (as verified by ToF-SIMS and EFTEM analyses).

Despite the fact that the solution was initially saturated with respect to amorphous silica, the ISG glass still corrodes. This phenomenon occurred under very stable conditions: pH was kept constant (Fig. 1a) and the Si concentration did not change for the entire duration of the test (Fig. 1b). In these conditions, the glass dissolution rate, given by the release time of B, Na or Ca (the three elements behave similarly), dramatically diminished during the experiment (Fig. 1c,d). The rate decrease is ~3.7 orders of magnitude from the first hours (~500 nm d^−1^, which is close to the maximum rate measured far from equilibrium^15^), to 363 days (around 0.1 nm d^−1^). In the meantime, Si, Al and Zr remained almost undissolved (at 363 days, the normalized loss of Al and Zr are 100 and 1,300 times smaller than that of B, respectively) (Supplementary Table 1). In addition, as the ^29^Si/^28^Si ratio remained stable over time, it could be concluded that ^29^Si_(aq)_ did not—or if so, only weakly—interact with Si from the glass during corrosion (Fig. 1b). From this, it seems that the three mobile elements behaved independently from the three low-soluble cations constituting the glassy network. This might seem at odds with the most of accepted models that attributed the drop in the corrosion rate to an increasing activity of the dissolved silica^16^,^17^,^18^,^19^.

### Mobile species display local gradients

**Figure 2:**
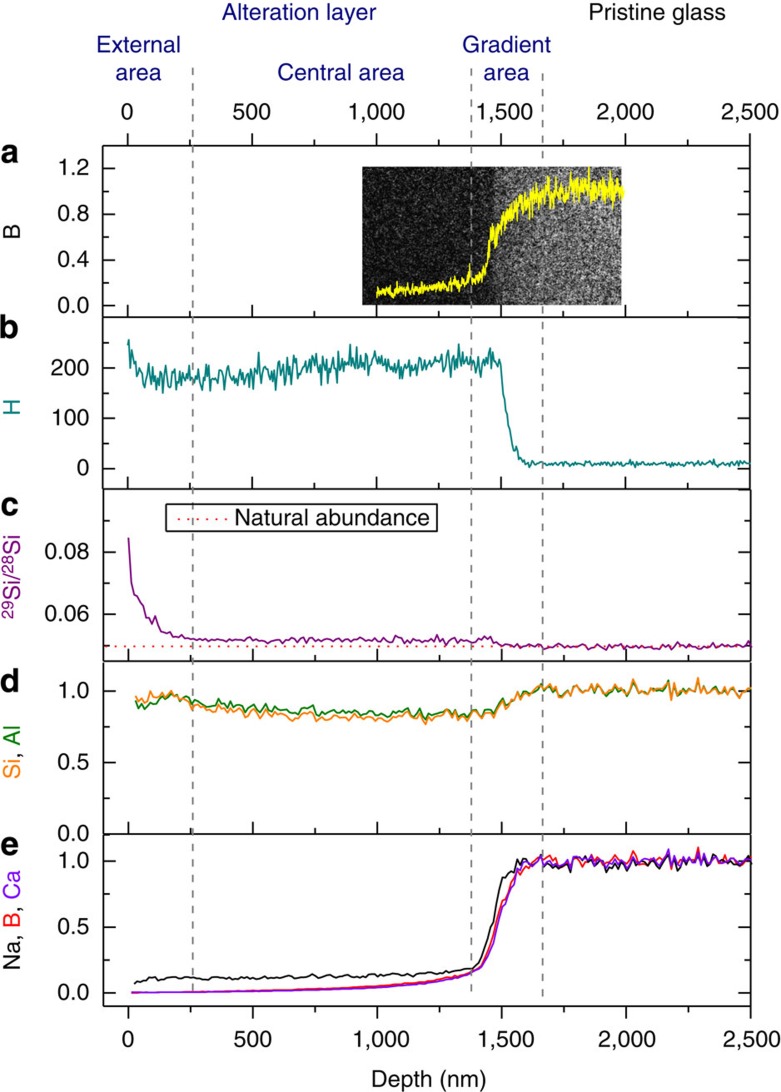
Elemental profiles within the 209-day sample. (**a**) EFTEM mapping and the superimposed profile of B concentration (normalized to that of the pristine glass). (**b**) The H profile (not-normalized) displaying an anticorrelated steeper gradient than that of the other mobile species of the glass (B, Ca and Na). (**c**) ^29^Si/^28^Si ratio exhibiting a slight enrichment in the first 250 nm of the alteration layer (this depth is possibly overestimated according to the caveat given in Methods) and then a constant value a few percent above the natural abundance (1(±1)% at 7 days, 5(±1)% at 209 days and 4(±1)% at 363 days). This ratio has to be compared with that of the bulk solution (~30). (**d**,**e**) Normalized profiles of sparingly soluble glass formers (Si and Al) and soluble elements (Na, Ca and B). Si and Al remain at a near constant concentration in the alteration layer, whereas mobile species (B, Ca and Na) are leached out of the layer except near the reaction front, where a gradient is clearly visible. Note that K is highly present in the alteration layer, but it is not displayed because its signal was saturated.

Deeper insights into the glass corrosion mechanisms were obtained from a detailed characterization of the altered glass coupons withdrawn at 7, 209 and 363 days. For the three samples, the alteration layer displays three sublayers (Fig. 2). From the solution to the pristine glass a *gradient area* can first be noticed, which B, Na and Ca concentrations dropped similarly. As time-of-flight secondary ion mass spectroscopy (ToF-SIMS) is known to potentially broaden chemical profiles especially in the case of rough or tilted interfaces^20^, B profile was also characterized by transmission electron microscope (TEM) in order to avoid misinterpretation. Energy-filtered TEM (EFTEM) B mapping performed on a focused ion beam cross-section prepared with the 209-day sample confirmed the position and the width of the B profile given by ToF-SIMS (Fig. 2a). ToF-SIMS also revealed that B, Na and Ca gradients are well anticorrelated with that of H (Fig. 2b and Supplementary Fig. 2). This pattern is generally attributed to ion exchange between exogenous positively charged species (H^+^, H_3_O^+^ and K^+^) and Na^+^, Ca^++^ from the glass and accompanied by the fast hydrolysis of B–O bonds^21^,^22^. Beyond this gradient area, B and Ca concentrations slowly decreased to zero, whereas Na remained at a low but constant concentration, likely to act as a charge compensator along with K for fourfold coordinated Al species and sixfold coordinated Zr species^23^. This large, *central area* showed a nearly flat concentration of all the glass constituents. Finally, an *external area* is visible, mainly characterized by significant changes in Si isotopic ratios (Fig. 2c).

### The alteration layer is not a precipitate

In the *central area* region the ^29^Si/^28^Si ratio plateaus at a few percent above the natural abundance (Fig. 2c). This slight enrichment of Si species supplied by the solution increased and stayed constant between 209 and 363 days. At the steady state, it represents one atom of Si supplied by the solution per 600 coming from the glass. This result suggests that the central part of the alteration layer is not a diffusion barrier for Si_(aq)_ as diffusion-limiting process would have led to chemical gradients. Therefore, one can assume that a thermodynamic equilibrium between the pore solution and the silicate network was achieved. The *external area*, ~250-nm thick (or less, according to the caveat given in the Methods section) showed a significant increase in the ^29^Si/^28^Si, especially in the first nanometre (Supplementary Fig. 3) where the isotopic ratio goes up to 1. Moreover, the profiles of the outermost sublayer are similar at 7 and 209 days (Supplementary Fig. 3). The latter observations strongly suggest that the glass surface underwent dissolution and reprecipitation reactions, and that an equilibrium between the external surface of the glass and the bulk solution was achieved within a few days. Overall, these findings are consistent with those obtained in a far more diluted medium, favouring the hydrolysis of Si–O–M bonds (M=Si, Al and Zr), where the whole alteration layer was significantly and gradually enriched in Si supplied by the solution^24^. Here except at the extreme surface, almost none of the silicon atoms of the glassy network were completely hydrolysed. These observations lead to two conclusions: first, a thermodynamic equilibrium is achieved between the surface of the altered glass and the bulk solution despite the absence of isotopic equilibrium inside the porous material; second, the altered glass cannot result from the congruent dissolution of the glass. The properties of the alteration layer next needed to be determined, to better understand the behaviour of the mobile species.

### The alteration layer acts as a molecular sieve

**Figure 3:**
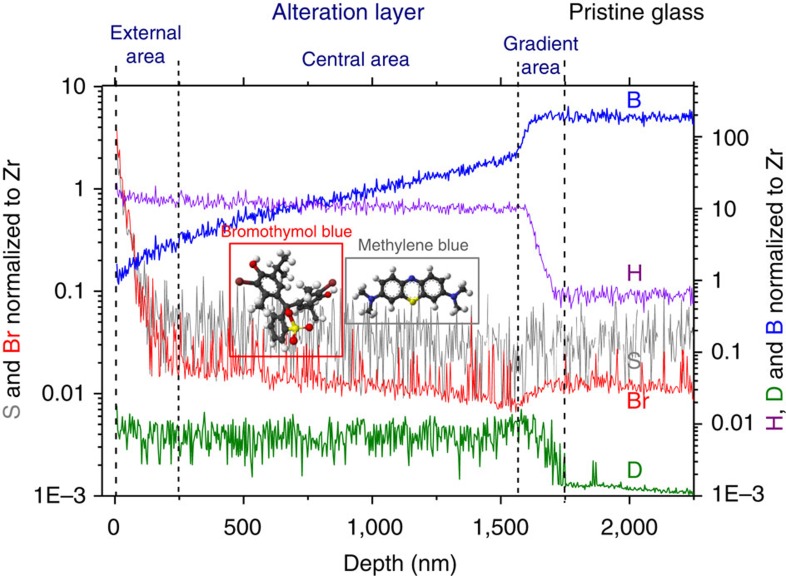
Elemental profiles within the 209-days sample after the tracing experiment with D_2_O and dyes. The sample spent 100 h at room temperature in contact with these molecules before ToF-SIMS depth profiling. S, Br and D are proxies for methylene blue (S in grey), bromothymol blue (Br in red) and D_2_O (D in green) molecules, respectively. B and H are displayed as reminders of the location of the different sublayers.

It has long been thought that Si-rich amorphous alteration layers formed during glass or mineral corrosion could be passivating^25^,^26^,^27^,^28^. However, the location and the mechanism limiting the transport of reactive species remained unclear. A post-experiment tracing test carried out with the 209-day glass sample showed that exogenous molecules added to solution at the end of the experiment behaved differently depending on their size (Fig. 3). Deuterium (0.27 nm in diameter) diffused easily up to the gradient area, and then its concentration dropped similarly to that of H, whereas the two dye molecules (1 nm in diameter) could only enter the first 250 nm of the alteration layer. As a result, the central part of the alteration layer acted as a molecular sieve with a cutoff <1 nm. According to the literature, such narrow channels have a huge impact on the fluid properties within the alteration layer^29^: the density, dielectric constant, activity and diffusivity of water are expected to significantly differ from that of the bulk, as well as phase solubility and ion hydration.

### The growth of the alteration layer affects the transport of aqueous species

**Figure 4:**
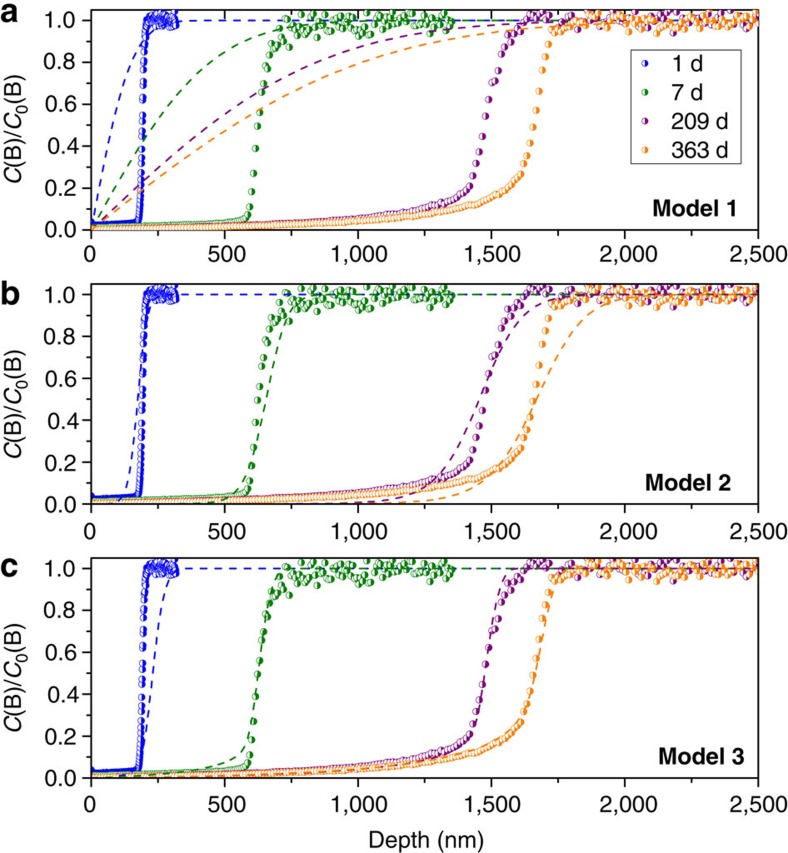
Time evolution of boron profile. Normalized B concentrations obtained with ToF-SIMS depth profiling (circles) are compared with simulations (dashed lines). The widths of the gradient area are 25 nm at the first duration, 150 nm at 7 days and 200 nm at the two last durations. (**a**) Comparison with Model 1, the simplest interdiffusion model, assuming a semi-infinite medium with a constant diffusion coefficient *D* of 6 × 10^−19^ m^2^ s^−1^. (**b**) Comparison with Model 2, a refined interdiffusion model assuming that water diffuses more slowly than mobile species from the glass. Here *b*=40,000 and *D*_w_=10^−20^, 2 × 10^−20^, 3.3 × 10^−21^ and 2.5 × 10^−21^ m^2^ s^−1^ at 1, 7, 209 and 363 days, respectively. (**c**) Comparison with Model 3, an advanced model with *D* spatially depending on the water concentration. Fits were obtained with *D*_max_=7.6 (±4.3) 10^−19^ m^2^ s^−1^, *α*=1.87 (±0.23), *β*=4.63 (±0.48).

The B profiles determined by ToF-SIMS (Fig. 4) display complex shapes that cannot be explained by classical interdiffusion models (Models 1 and 2 in our case)^30^,^31^,^32^. Although Model 2 fits better with our experimental data than Model 1, it must be considered that the diffusion coefficients of water and B diminish with time. Moreover, the shape of the B profiles is not well fitted with Model 2. However, simple considerations can be taken into account. The initial diffusion coefficient of mobile species of the glass through the newly formed alteration layer, *D*_ma*x*_, was derived from the first day of alteration of the 4- to 6-μm glass powder placed in the same conditions as the glass monoliths and studied by solid-state nuclear magnetic resonance (NMR) spectroscopy. According to the corresponding value of *D*_max_ of 6 × 10^−19^ m^2^ s^−1^, a glass alteration thickness of 5.3 μm after 363 days was expected, a value three times greater than the actual thickness. This result confirms the hypothesis derived from Model 2, suggesting that the apparent diffusivity of mobile species became lower than at the beginning. To explain this effect, a third model was developed (Model 3). Following this model (Fig. 4c), *D* is expected to drop by 4–5 orders of magnitude between the outermost layer and the reaction front, to account for the peculiar shape of the B profiles. The reasonable good quality of the fit provided by this model, with low variation of the fitting parameters, suggests that the transport of mobile species is space-dependent explaining why it was suggested to be time-dependent. *D* could thus be positively correlated to a local concentration of the chemically rate-limiting species, probably one of the water species. A strong hypothesis to account for this phenomenon, although never investigated in silica-saturated conditions, is the reorganization of the silicate network following the departure of the mobile species^22^,^33^,^34^. This is the aim of the following investigations.

### Silica does not polymerize in solution

**Figure 5:**
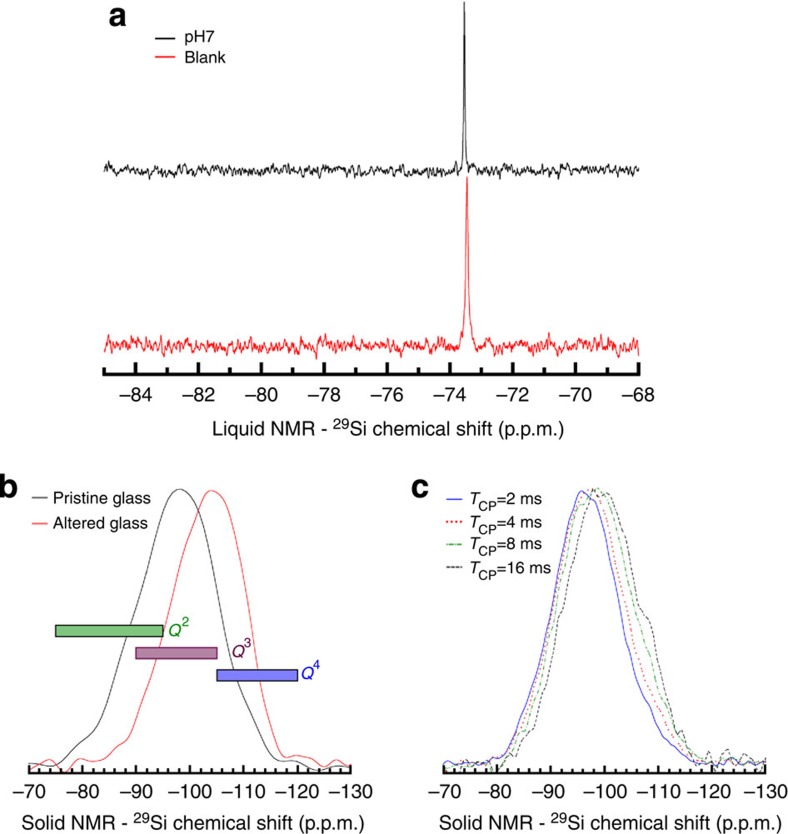
Liquid-state and solid-state ^29^Si NMR spectroscopy performed both on solution and on pristine and altered glass. (**a**) Leaching solution at the end of glass alteration (pH 7) compared with the initial ^29^Si-enriched solution (Blank). (**b**) Pristine and fully altered glass from MAS NMR and (**c**) fully altered glass from CP-MAS NMR, obtained with various proton-to-silicon magnetization transfer times (*T*_CP_) from 2 to 16 ms. *Q*^*n*^ refers to the degree of connectivity of a SiO_2_ tetrahedron, where *n*=0–4 corresponds to the number of bridging oxygen atoms.

Although Si_(aq)_ is poorly reactive with corroding glass, we investigated both its speciation in solution and the structure of the alteration layer by liquid-state and solid-state ^29^Si NMR spectroscopy. Figure 5a shows only one species of Si_(aq)_ in the initial solution and in the leachate, which corresponds to monomers (H_4_SiO_4_)^35^. Therefore, polymerization reactions that could be expected^36^ in these highly concentrated solutions did not occur. As a result, it can be supposed that Si_(aq)_ species are small enough (~0.5 nm) to freely diffuse within the alteration layer up to the gradient area. This assumption is in agreement with the slight homogeneous increase in the ^29^Si/^28^Si ratio in the central area of the altered layer (Fig. 2c).

### *In situ* reorganization after silicate network corrosion.

Solid-state ^29^Si MAS NMR spectroscopy was performed on fully altered grains of glass corroded under similar conditions as the monoliths. Owing to their different electronegativity, the main glass-former cations Al, ^IV^B and Zr increase the chemical shift, *δ*_Si_ (refs 37, 38, 39, 40), whereas Ca gives an opposite effect because of its location near the nonbridging oxygen atoms^41^. The Carr Purcell Meiboom Gill (CPMG) sequence^42^ as displayed in Fig. 5b indicates a negative shift of *δ*_Si_ from pristine to altered glass. In a first approximation, this shift could be an artifact tied to the release of B. However, because of the opposite effect of Ca, and the low proportion ^IV^B compared with Si, the observed shift is more likely to be attributed to a higher degree of polymerization of the alteration layer compared with the glass. This hypothesis is confirmed by cross-polarization ^1^H-^29^Si NMR analyses (Fig. 5c). By increasing the magnetization transfer time, larger distances between protons and Si were probed. Excitations of species with high numbers of bridging atoms to Si (SiO_4_) are then at the origin of the *δ*_Si_ shifts to lower values. To our knowledge, this is the first direct evidence of the reorganization of the silicate network at high reaction progress. This reorganization was not expected based on the chemical analyses of the Si behaviour both in solution and in the altered glass. From a structural point of view, it may be in agreement with Molecular Dynamic and Monte Carlo simulations, which showed that the hydrolysis of B–O–Si bonds, the release of Na and Ca from nonbridging oxygen atom, and the potential hydrolysis of some Si–O–Si bonds first form Si–OH groups that can then condense to reform Si–O–Si bonds^21^. This *in situ* reorganization is likely to be less energy-consuming than the complete hydrolysis of the glass species and their precipitation into more stable phases.

## Discussion

Two visions of the rate-limiting mechanisms are currently under discussion in the literature. On the one hand, glass is expected to dissolve congruently in a nanometre-size film of water, and dissolved species precipitate from the liquid phase^43^,^44^,^45^,^46^. On the other hand, glass undergoes ion exchange, whereby positively charged water species replace alkalis in glass-modifier position with partial or no dissolution of the remaining silicate network once silica saturation is achieved in the bulk solution^18^,^22^,^47^. Our findings show that none of these paradigms are strictly correct at least for explaining the rate under silica saturation conditions. The release of mobile cations from the glass is associated with a high repolymerization of the silicate network leading to a self-reorganized submicroporous material, in which only small molecules (<1 nm) can diffuse. Preliminary data suggest that the water content of this material is very low (~3 wt%) compared with porous gels formed far from saturation (~ 10–20 wt%). Moreover, the most of this material is passivating, as evidenced by a significant decrease in the mobile species diffusivity. The removal of B, Na and Ca from the glass deeply affects the structure and the properties of the alteration layer, triggering the repolymerization of the silicate network and the resulting material in turn affects the release of mobile species. In addition, it could be noted that the porosity clogging of the external part of the alteration layer, as demonstrated in other studies^48^,^49^, is not a prerequisite for the rate drop. It happens for some glasses, making them more durable, but in most cases the porosity stays open as shown here and the passivation occurs with the growth of the self-reorganized alteration layer^9^. The reason why *D* drops, making the glass/alteration layer interphase more efficient than the glass itself in preventing water ingress, may now be discussed. On the basis of thermodynamic and kinetic considerations, at least three hypotheses seem possible. (1) The water mobility and reactivity within the alteration layer are lower than in the bulk solution. This hypothesis is supported by the extremely small pore size of the alteration layer (<1 nm) accounting for the fact that there are no free water molecules in this medium^50^. (2) The flux of water towards the glass drops along with the growth of the alteration layer. (3) Liquid and solid phases within the reacting interphase approach a global thermodynamic equilibrium. Model 3, successfully applied to our experimental results, cannot help rank the previous hypotheses. Nonetheless, the results strongly suggest that transport of elements and chemical reactivity of nanoconfined water are closely linked^51^. It is worth noting that the diffusivity of He—a chemically inert atom with the same size as a water molecule—in this type of glass is ~10^−15^ m^2^ s^−1^ at 90 °C (ref. 52), a value 4–9 orders of magnitude lower than water diffusivity in pristine and altered glass. This huge difference supports the idea that water mobility in pristine and altered glass is strongly affected by chemical interactions with the solid phase. These reactions need to be better understood.

These findings give rise to several points. First of all, glass corrosion mechanisms—especially those controlling the long-term behaviour of glass in confined medium—are better understood. As the literature reports strong mechanistic analogies between nuclear and natural glasses^53^, the long-term behaviour of basaltic glass in confined environments could be re-evaluated. For the first time, it has been demonstrated that under silica saturation conditions, (i) the reorganized alteration layer achieves equilibrium with the bulk and pore solutions and (ii) the residual corrosion rate dramatically diminishes due to transport-limiting effects near the glass surface. On the conservative assumption that boron release rate stops diminishing, the time required to corrode 1 cm of glass at a rate of 0.1 nm d^−1^ (the rate obtained at 363 days, Fig. 1d) would exceeds 250,000 years at 90 °C. It is worth noting that most of the results presented in this paper have been confirmed at pH 9, although glass corrosion is slower and the resulting layers are more difficult to characterize because they are thinner. Hence, such conditions seem favourable to guarantee a very high durability of nuclear glass. According to our results and the large experimental database on nuclear glass corrosion, including industrial materials, these conditions could be extended to the following: *T*≤90 °C, 7≤pH≤9.5, silica-saturated solution and low renewal rate of the solution. The disposal of glass packages that are under consideration in many countries (for example, the USA, the UK, France, Japan, Belgium and Germany) could be optimized by meeting such conditions. It must, however, be kept in mind that other reactions not observed here can make the glass less durable. Among them, the most detrimental effect is the precipitation of silicate minerals (phyllosilicates, iron-silicates, zeolites, calcium-silicate-hydrates, and so on) that sustain glass corrosion by consuming Si_(aq)_ and potentially other glass formers from both the solution and the alteration layer and disrupting the passivating layer^6^,^54^,^55^,^56^. The mechanism is well known but only a few studies have attempted to model the competition between the amorphous layer and other minerals owing to the lack of knowledge on this nonstoichiometric material (composition, dissolution rate and solubility)^57^,^58^. Thanks to the methodology and the protocols proposed here, our study opens the way to further investigations and applications.

## Methods

### Glass preparation

The ISG glass (60.2 SiO_2_, 16.0 B_2_O_3_, 12.6 Na_2_O, 5.7 CaO, 3.8 Al_2_O_3_ and 1.7 ZrO_2_ in mol%) was produced in 2012 by MoSci Corporation (Rolla, MO, USA). The glass was melted in platinum–rhodium crucibles in an electric furnace at 1,300 °C for ~4 h, stirred once with a quartz rod and cast into a graphite mold. The ingots were annealed at 569 °C for 6 h in an electric oven and cooled to room temperature at a rate of 50 °C per hour. Sixteen coupons of 2 × 2 × 0.11 cm^3^ each with only one large face polished were put in polytetrafluoroethylene holders and placed at 90 °C in a perfluoroalkoxy vessel containing 380 ml of solution initially saturated with amorphous ^29^SiO_2_ (*C*(Si)=141 mg.l^−1^;Supplementary Fig. 1). Note that glass coupons were prepared with one face polished and the other let raw after being cut with a diamond saw in order to see how fast rough surfaces become smooth due to corrosion. This question is partly addressed in a similar study conducted at pH 11.5 (ref. 13). To obtain the starting solution, ^29^SiO_2_ (Eurisotop,^29^Si>95%) was mixed with KOH (Suprapur) and melted at 600 °C. The resulting potassium silicate was then dissolved in deionized water. This protocol led to a concentration of K of 6.8 g l^−1^. It was verified separately that the concentration of K has no influence on the glass corrosion behaviour. The pH_90 °C_ was maintained at 7±0.25 throughout the experiment with regular additions of HNO_3_ 10^−2^ M. The reactive surface area of a glass coupon was estimated at 15±2 cm^2^ by measuring the ‘not-normalized’ initial dissolution rate at pH 9 (in g d^−1^) of a glass coupon similar to those utilized in this study and then dividing this rate by the normalized dissolution rate (in g m^−2^ d^−1^) obtained in the same conditions with a fully polished coupon. More details are given in ref. 13.

For the solid-state NMR analysis, 300 mg of small 4- to 6-μm-diameter particles of the same glass were prepared by milling and decantation in acetone. This powder was altered under similar conditions (90 °C, pH_90 °C_ 7, *C*(Si)=135 mg l^−1^) until the complete release of B.

### Solution analysis

The solution was regularly sampled and cations were analysed by inductively coupled plasma-optical emission spectroscopy (Thermo Scientific, ICAP 6300 DUO). Silicon isotopes were analysed by multicollector inductively coupled plasma—mass spectroscopy (Nu Instruments Nu Plasma HR). The equivalent thickness of altered glass, *ETh*, was calculated from B concentration at a sampling duration *t* as follows:


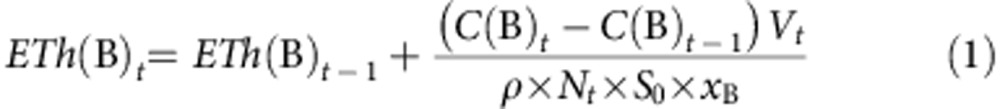


where *C*(B) is the B concentration, *V*_*t*_ the solution volume at the time *t*, *ρ* the glass density (2.50 g cm^−3^), *N*_*t*_ the number of glass coupons still present in the vessel at the time *t*, *S*_0_ the reactive surface area of a glass coupon (15±2 cm^2^) and *x*_B_ the mass fraction of B in the glass (0.0539). The glass dissolution rate *r* is given by:


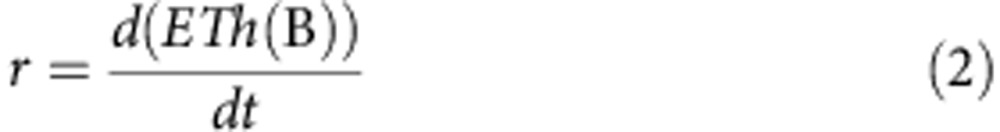


The uncertainties for *ETh*(B) and *r* are 10% and 30%, respectively.

### Post-experiment tracing test

At three durations (7, 209 and 363 days) a glass coupon was withdrawn, rinsed and cut into four pieces before characterization. One piece of the 209-day sample was immersed 100 h at room temperature in a solution at pH 7, saturated with SiO_2am_ and spiked with 1.2 mol l^−1^ D_2_O, 8.0 10^−5^ mol^−1^ bromothymol blue and 4.4 10^−4^ mol l^−1^ methylene blue.

### Depth profiling analyses

The 209-day glass coupon before and after immersion in the spiked solution, along with the two coupons collected on 7 and 363 days and a last one (0.5 × 0.5 × 0.2 cm^3^) added to the solution at the end of the experiment and withdrawn 1 day after, were analysed by ToF-SIMS (IONTOF TOF 5). Two sputtering beams, analysing a surface area of ~60 × 60 μm^2^, were utilized: Cs^+^ (for D, H, Br and S) or O_2_^+^ (for the other elements). The oxygen beam was either set at 2 keV, 545 nA to quickly analyse the whole alteration layer and some pristine glass, or at 500 eV, 100 nA to get more accurate data of the external part of the alteration layer. It must be noted that the first analytical conditions provide a lower depth resolution (~12 versus ~0.1 nm per step) and also tend to shift the profiles of species weakly bonded to the network because of energetic collisions with O_2_^+^ ions (the case of ^29^Si atoms, which have diffused from the bulk solution, as well as Br and S). Elemental profiles were normalized to that of Zr to avoid matrix effects. For the glass components *i* (*i*=Si, B, Na, Al, Ca), the ratios *i*/Zr were then normalized to that measured in the pristine glass.

### Modelling B profiles

Boron profiles were fitted according to three models. Model 1 assumes that the glass is a nonreactive, semi-infinite medium with a planar, nonstationary (that is, retreating) fluid–solid interface. The normalized concentration of B, resulting from the resolution of the second Fick’s law, is given by^59^:


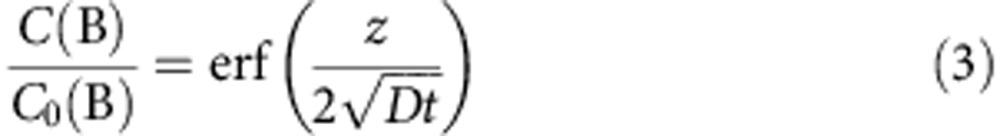


where *C*(B) and *C*_0_(B) are the concentrations of B at a given depth *z* and in the pristine glass, respectively, *D* is the apparent diffusion of B—a constant parameter in this model—and *t* the time.

Model 2 is based on Doremus’ model assuming that water diffuses more slowly than B^32^. The normalized concentration of B becomes:


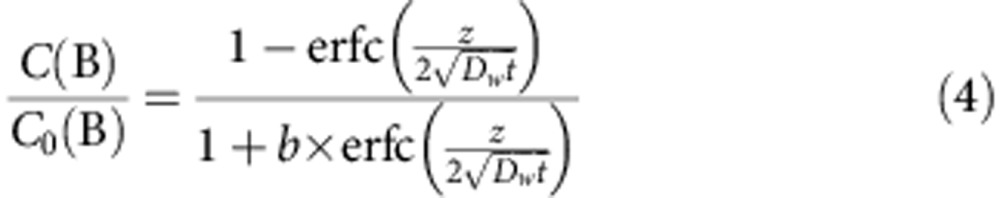


where *D*_w_ is the diffusion coefficient of water, *b*=*D*/*D*_w_−1.

Unlike Model 2, Model 3 assumes that *D* depends on local concentration of water, a likely rate-limiting species. As water replaces mobile species released by the glass, it is assumed in a first approximation that the concentration of water is inversely proportional to the B concentration. Equation 4 is postulated to account for *D* variations with local boron concentrations.


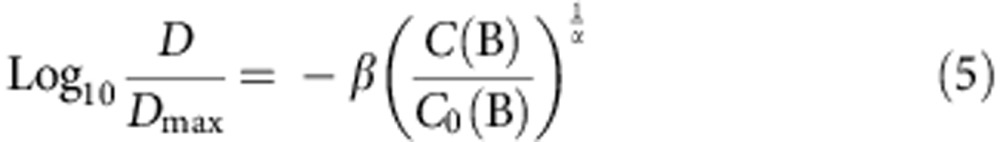


where *D*_max_ corresponds to the maximal value of the apparent diffusion of B in the alteration layer, while *α* and *β* are two fitting parameters. For *β*=1, *D* is 10 times lower than *D*_max_, 100 times in case *β*=2. Parameter *α* describes how *D* drops with increasing B concentration (decreasing water concentration). The value of *D*_max_ (6 × 10^−19^ m^2^ s^−1^) was approximated by measuring the release of mobile species during the first stage of glass corrosion, when preparing the powder for the NMR study. The second Fick’s law, in which *D* variations are given by Equation 5 was solved numerically following a finite element approach.

### Electronic microscopy

Scanning electron microscope (SEM) observations as well as TEM analyses (energy-dispersive X-ray spectroscopy and EFTEM) were also performed on the 209-day sample to confirm our results. For the TEM study, a thin film of ~100-nm thick was prepared with a dual beam SEM/FIB microscope (FEI Helios NanoLab 600) following the method described in ref. 60. EFTEM boron images were acquired with a TEM FEI Titan 80–300 Cs operating at 200 kV under the following conditions: acquisition time 10 s per image, energy window width 20 eV and a bining of 2 × 2, giving 1,024 × 1,024 pixel images. Images were processed with Gatan’s Digital Micrograph software package. The three-window technique was used to obtain boron elemental maps^61^. The B profile was calculated with the ImageJ software.

### NMR spectroscopy

Liquid-state ^29^Si NMR spectra were recorded on a 400-MHz Agilent DD2 spectrometer equipped with the OneNMR 5-mm probe and without sample-spinning, following the procedure described elsewhere^13^. ^29^Si cross-polarization magic-angle spinning (CP-MAS) NMR spectra were collected on a Bruker Avance 300 MHz spectrometer (magnetic field 7.05 T) using a 4-mm (rotor outer diameter) Bruker CP-MAS Probe and sample-spinning frequency of 10,000 Hz. CPMG ^29^Si MAS spectra collected are described in ref. 40, co-adding typically 20–40 echoes, with a recyle delay of 20 s (no changes in lineshape were observed between 2 and 200 s). CP-MAS ^29^Si{^1^H} spectra were collected with a recycle delay of 2 s and ^1^H-^29^Si magnetization transfer ranging from 0.5 to 16 ms.

### Consistency of the measurements

Consistency of the glass alteration thickness measurements was verified using four independent techniques. It was found that all the measurements are consistent within a range of ±15% (Supplementary Fig. 4).

## Author contributions

S.G. supervised the study and wrote the paper. P.J. and M.F. were deeply involved in the interpretation. P.F. modeled the B profiles. F.A. and T.C. performed the solid-state NMR study.

## Additional information

**How to cite this article:** Gin, S. *et al.* Origin and consequences of silicate glass passivation by surface layers. *Nat. Commun.* 6:6360 doi: 10.1038/ncomms7360 (2015).

## Supplementary Material

Supplementary Information Supplementary Figures 1-4 and Supplementary Table 1
