# Supplementary material for: Origin and consequences of silicate glass passivation by surface layers
Source: Nat Commun. 2015 Feb 19;6:6360. doi: 10.1038/ncomms7360 (PMC4346618; doi:10.1038/ncomms7360)
Supplement: Supplementary Information — Supplementary Figures 1-4 and Supplementary Table 1 [file ncomms7360-s1.pdf]

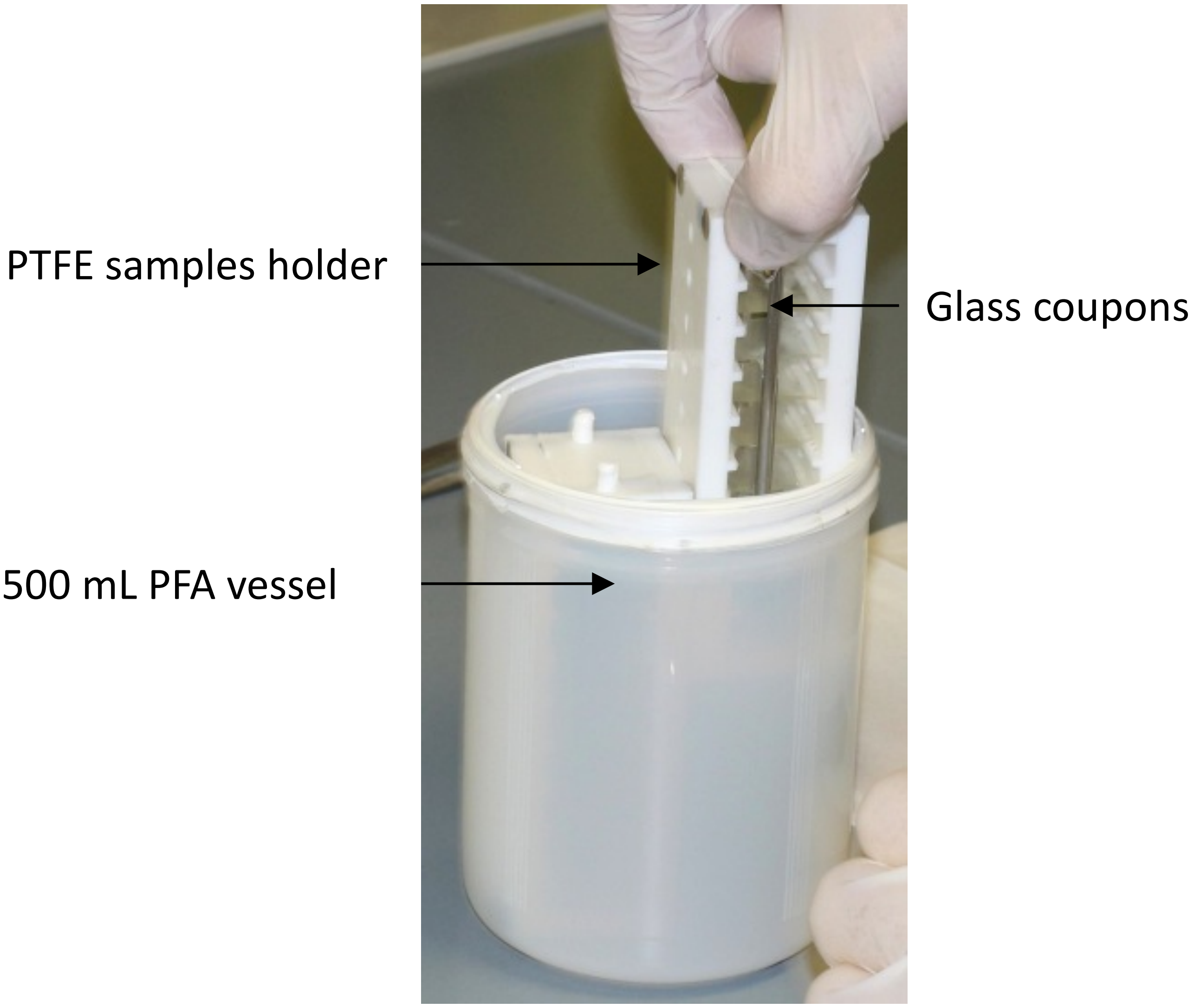

Supplementary Figure 1. Experimental set up for the isotopically tagged experiments. Glass coupons are mounted on two PTFE holders. The reactor was filled with 380 mL of solution and closed tightly. In order to reduce evaporation below 1 mL/month, the reactor was placed in a larger PFA vessel containing a small amount of deionized water. The experiment was performed for 1 year at 90°C.

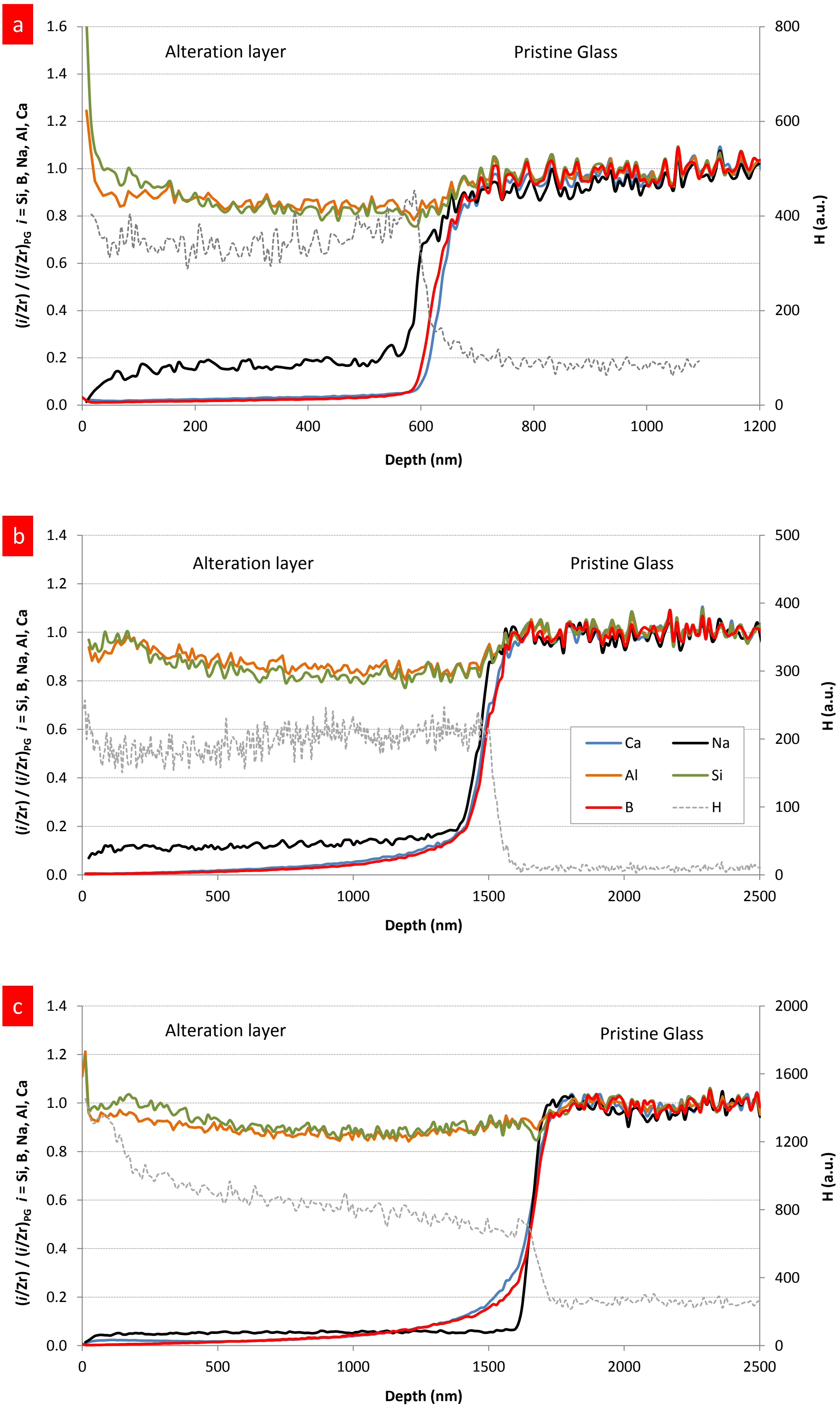

Supplementary Figure 2. ToF-SIMS profiles at 7 days (a), 209 days (b) and 363 days (c). Glass components (Si, B, Na, Al, Ca, Zr) were analyzed as positive ions following sputtering with O_2^+ beam. Their profiles are normalized. H was analyzed separately, as negative ions following sputtering with Cs^+ beam. Their profiles are not normalized (a.u. stands for arbitrary unit).

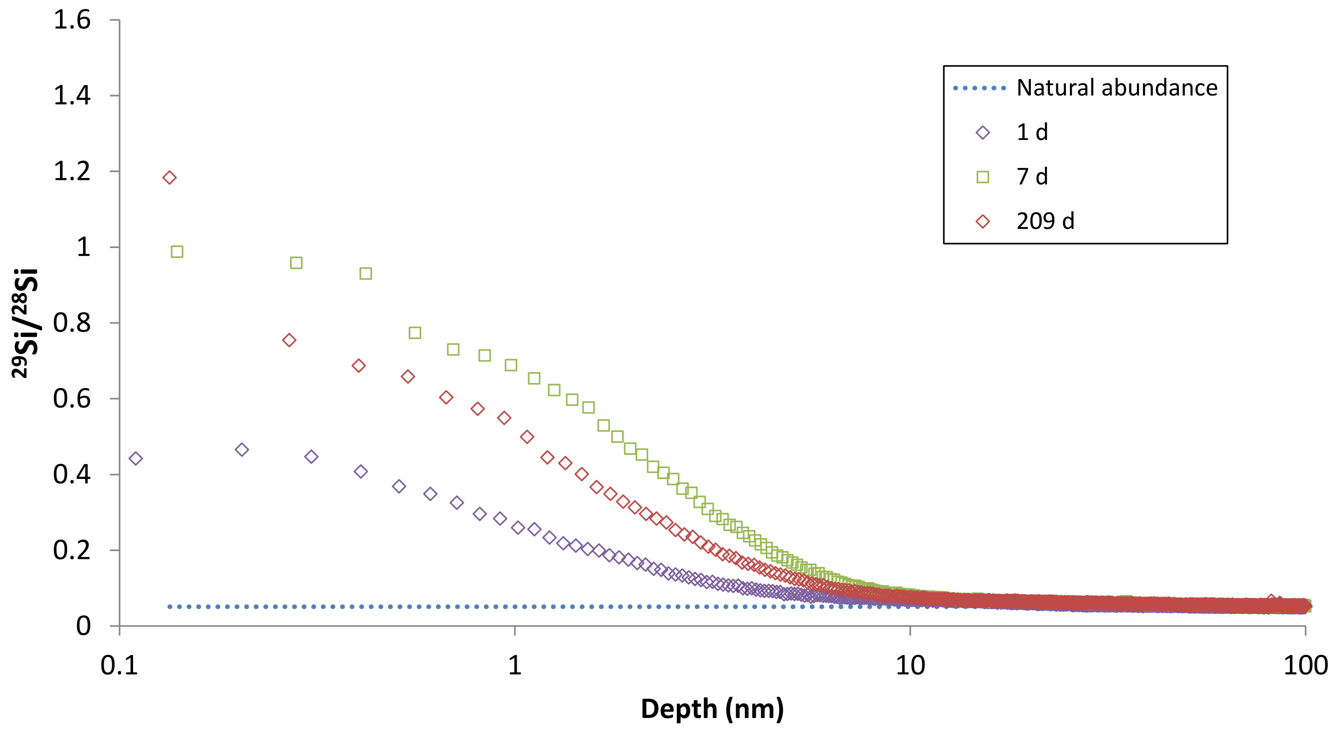

Supplementary Figure 3. ToF-SIMS profiles of $^{29}Si/^{28}Si$ within the first hundred nm of the glass alteration layer formed at 1, 7 and 209 days. For these analyses, the sputtering rate of the altered glass was minimized in order to decrease the analytical step up to about 0.1 nm.

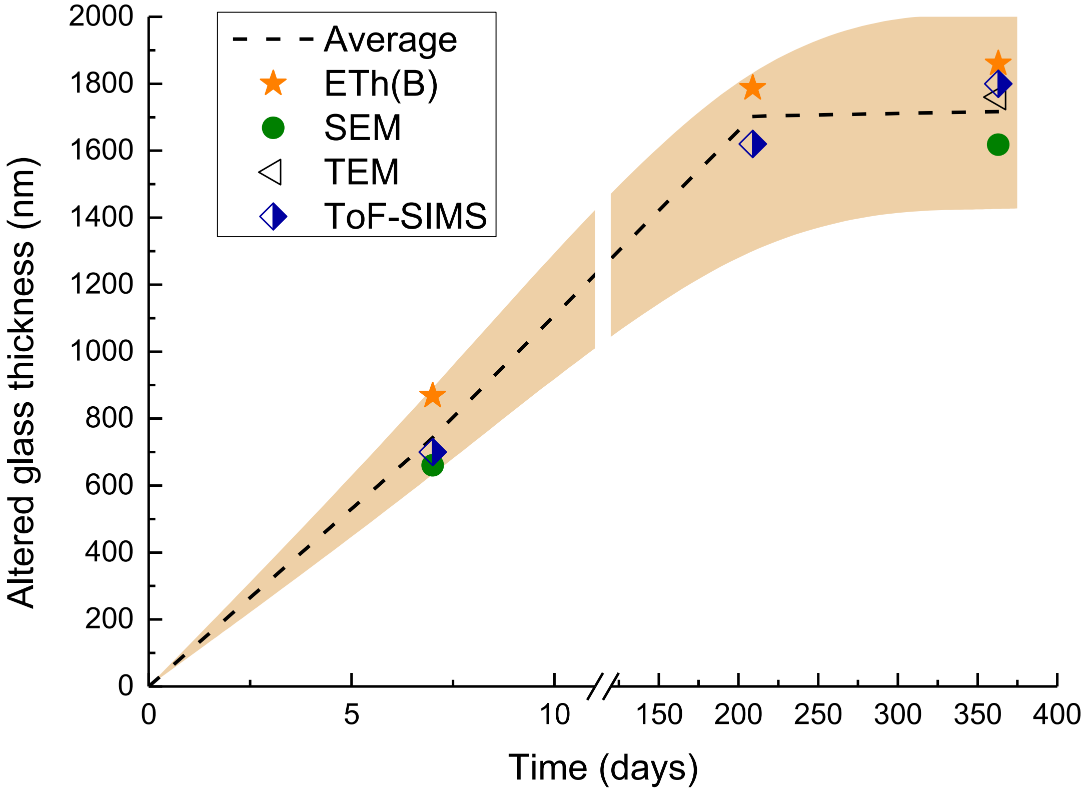

Supplementary Figure 4. Consistency of independent measurements of the altered glass thicknesses performed at 7, 209 and 363 days. The solid line corresponds to a linear interpolation between mean values calculated at each duration and the beige colored area between dashed lines delimits a deviation of ± 15% around the mean curve. The symbols correspond to 4 independent measurements of the glass alteration thickness: B release to solution, cross sectional SEM observation of the alteration layer, TEM observation of the alteration layer from a FIB preparation and ToF-SIMS depth profiling. All the measurements match within 15% of uncertainty.

			ICP-OES						MC-ICP-MS							
t days	$pH_{90°C}$	AGF %	Si mg L^{-1}	B mg L^{-1}	Na mg L^{-1}	Al mg L^{-1}	Ca mg L^{-1}	K g L^{-1}	^{28}Si mg L^{-1}	^{29}Si mg L^{-1}	^{30}Si mg L^{-1}	Si tot mg L^{-1}	$^{29}Si/^{28}Si$	Al mg L^{-1}	Ca mg L^{-1}	Zr µg L^{-1}
0	7.01	0	141.1	<0.2	<1	<0.2	<1	6.8	3.4	116.9	2.7	123.0	32.7	nd	nd	nd
0.3	7.30	<0.1	139.5	0.81	3.1	<0.2	2.5	6.7	3.2	117.0	2.8	123.0	35.4	nd	nd	nd
7	7.08	0.2	139.4	5.5	11	<0.2	-	6.7	2.6	111.5	2.6	116.7	34.5	nd	nd	nd
14	7.13	0.3	138.8	7.0	9.3	<0.2	-	6.7	3.0	121.4	2.8	127.3	39.8	nd	nd	nd
28	7.12	0.4	140.5	8.7	19	<0.2	6.1	6.8	3.5	116.2	2.7	122.5	32.4	nd	nd	nd
91	7.20	0.5	141.2	10.4	35	<0.2	5.6	6.9	3.2	115.0	2.7	120.9	35.0	nd	nd	nd
209	7.12	0.5	140.3	10.8	-	<0.2	-	6.9	2.8	91.5	2.1	96.5	31.1	nd	nd	nd
273	7.17	0.5	140.2	11.0	-	<0.2	-	6.6	3.0	103.1	2.4	108.5	32.9	0.08	8.2	2
363	7.27	0.5	140.2	11.0	-	<0.2	-	6.0	2.6	83.4	1.9	87.9	30.4	0.07	8.4	4

Supplementary Table 1. Data from solution analyses performed on the experiment at pH 7. The experiment was not repeated. **'**AGF' stands for Altered Glass Fraction, 'nd' stands for Not Determined and '-' means that the uncertainty exceeds 100%. Elemental concentrations measured by ICP-OES are given with an error of ± 3%, except for Na (± 50 % due to contamination by K-bearing products). High concentration of K comes from the preparation of the starting solution (silica was dissolved after alkaline fusion with KOH). Elemental concentrations measured by MC-ICP-MS are given with an error of ± 15% due to matrix effect. $^{29}Si/^{28}Si$ ratios are given with an error of ± 10%.
